# Case report: USG of bilateral tuberculous epididymo-orchitis

**DOI:** 10.4103/0971-3026.38508

**Published:** 2008-02

**Authors:** Baphira Wankhar, Prem P Batchala, Stephen Sailo

**Affiliations:** 1 Department of Radiodiagnosis and Imaging, North-Eastern Indira Gandhi Regional Institute for Health and Medical Sciences, Mawdiangdiang, Shillong, Meghalaya - 793 012, India; 2 Department of Radiodiagnosis and Imaging, Bethany Hospital, Shillong, Meghalaya - 793 003, India; 3 Department of Urology, North-Eastern Indira Gandhi Regional Institute for Health and Medical Sciences, Mawdiangdiang, Shillong, Meghalaya - 793 012, India

**Keywords:** Scrotum, testes, tuberculosis, ultrasound

The genitourinary tract is the most common extrapulmonary site affected by tuberculosis.[1] The male genital organs are involved in more than 50% of patients.[2] The epididymis is the commonest structure to be involved, followed by the seminal vesicles, prostate, testis, and the vas deferens.[3] Bilateral involvement of scrotal structures is not rare. Bilateral epididymal involvement has been reported in 25% of patients with scrotal tuberculosis.[4] We would like to report a patient with bilateral tuberculous epididymo-orchitis.

## Case Report

A 70-year-old man presented with bilateral scrotal swelling and mild, vague pain for 2 weeks. The patient also complained of bilateral flank pain and dysuria. Scrotal examination revealed thickened cords and nodular epididymides on palpation. Mild right renal angle tenderness was elicited. Laboratory investigations revealed an elevated ESR. Clinically there was a strong suspicion of chronic granulomatous inflammation.

**Figure 1(A-D):**
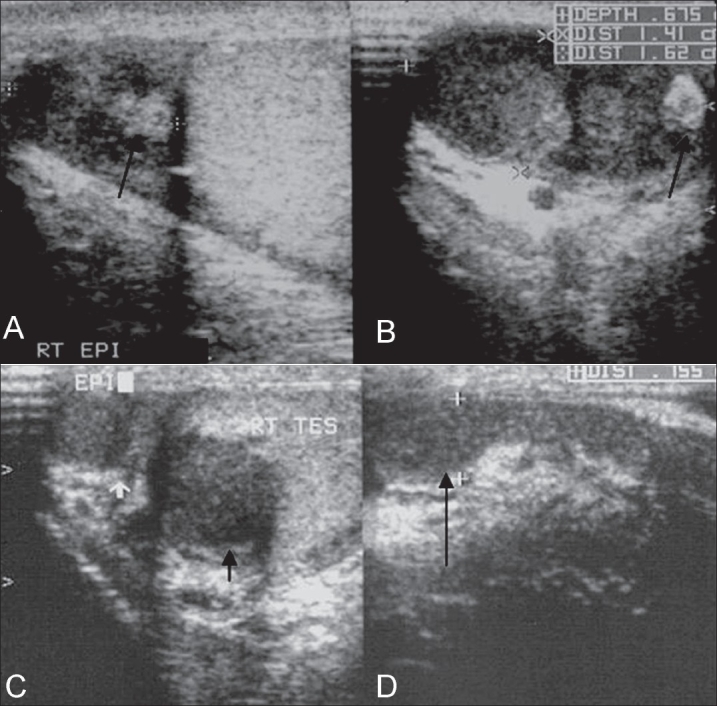
Longitudinal (A) and transverse (B) USG images of the right epididymal head show enlargement and heterogeneous hypoechoic echotexture. Calcification is seen as a hyperechoic nodular focus (black arrows). Transverse (C) and longitudinal (D) USG images of the right epididymal tail show a heterogeneous and hypoechoic tail (short white arrow in C and long black arrow in D). The hypoechoic right rete testes is also noted (short black arrow in C)

**Figure 2(A, B):**
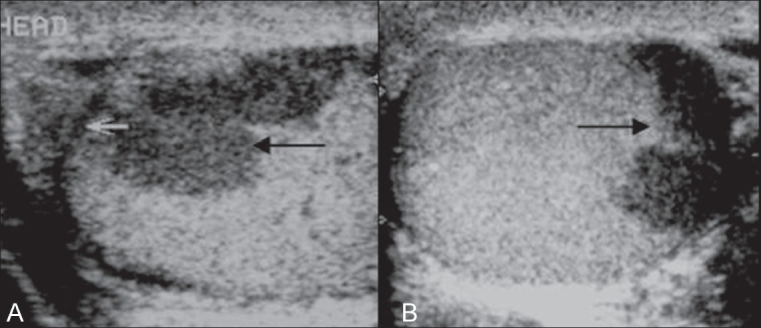
Transverse USG image (A) of the right testis shows a hypoechoic rete testis (black arrow) with an irregular geographic inner border. The hypoechoic right epididymal head is also seen (white arrow). The transverse image (B) of the hypoechoic left rete testis (black arrow), fairly similar to the right testis, is noted

**Figure 3:**
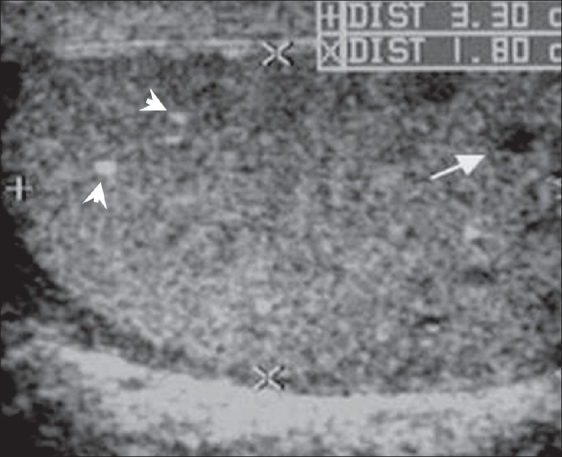
Transverse USG image shows a small hypoechoic nodule (white arrow) in the right testicular parenchyma with small, punctate areas of parenchymal calcification (arrowheads)

The chest radiograph was normal. USG of the scrotum revealed bilateral diffusely enlarged epididymides with a heterogeneous and predominantly hypoechoic echotexture [Figure 1]. There was nodular focal calcification seen in the right epididymal head [Figure 1]. These features were suggestive of chronic granulomatous inflammation, probably of tuberculous etiology. In addition, both testes showed symmetrically hypoechoic rete testes, posterior to the mediastinum (isoechoic and in contiguity with the involved epididymides), with irregular geographic inner borders - a finding not previously described in literature [Figure 2]. This finding represents contiguous extension of the granulomatous inflammation from the epididymides into the tubules in the rete testes. There were also a few, small, focal, rounded hypoechoic lesions within the testicular parenchyma [Figure 3]. A small hydrocele was present in the right scrotal sac.

**Figure 4(A-D):**
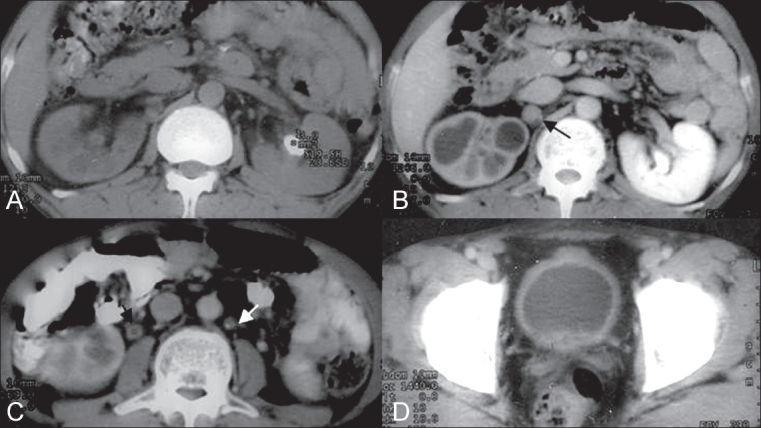
Noncontrast CT image (A) at the level of kidney shows a left renal calculus. A contrast-enhanced CT image (B) shows a hydronephrotic right kidney with mild parenchymal atrophy and a poor nephrogram, along with an enhancing retrocaval lymph node (black arrow). A contrast-enhanced CT image through the lower renal poles shows right ureteritis, with marked wall thickening (black arrow). A partially calcified retroperitoneal lymph node (white arrow) is also seen. A contrast-enhanced CT image at the level of the urinary bladder shows a thickened, enhancing bladder wall, suggestive of cystitis

Abdominal USG followed by CT scan showed a hydronephrotic right kidney with noncommunicating dilated upper pole calyces, suggesting an infundibular stricture. There was a large calculus in the left renal pelvis [Figure 4A]. The right kidney showed a poor nephrogram and poor contrast excretion [Figure 4B]. The right ureter also showed mild mural thickening with enhancement, suggesting ureteritis [Figure 4C]. In addition, multiple partially calcified lymph nodes were seen at the porta and in the retroperitoneum and mesentery [Figure 4B and C]. The urinary bladder showed mild wall thickening with enhancement, suggesting cystitis [Figure 4D].

Urine examination revealed pyuria but was negative for acid fast bacilli. Fine needle aspiration from the epididymal lesion was consistent with tuberculosis, showing caseating necrosis and epitheloid cells. Antituberculous therapy was instituted and a left nephropyelolithotomy was also planned to salvage the left kidney. However, this patient was lost to follow-up and no post-treatment details are currently available.

## Discussion

The route of entry of the tuberculous bacillus into the scrotal sac structures is a topic of controversy.[5] Most believe that tuberculous epididymo-orchitis is secondary to direct retrograde spread from the urinary tract via reflux. Though a recent retrospective study in 40 cases[6] revealed isolated tuberculous epididymitis, without evidence of renal involvement on urine examinations and intravenous pyelography, the possibility of a healed renal focus which had excreted the bacilli could not be ruled out. The tuberculous bacillus can also gain entry via the hematogeneous and lymphatic routes. With both direct and hematogenous spread, the tail of the epididymis is usually the first structure to be involved,[7–9] probably due to its greater vascularity.[10]

One interesting USG finding in our patient - a finding not described earlier in the literature on scrotal tuberculosis - favors the possibility of the retrograde direct route of spread: both rete testes were symmetrically heterogeneous and hypoechoic, with well-defined, geographic, irregular inner borders. Their echogenecity was similar to that of the involved epididymides and their involvement was continuous with the enlarged body and tail of the epididymides. With active tuberculosis in the upper urinary tract, a symmetrical finding such as this can very well be explained by the retrograde reflux theory. A few discrete, small, hypoechoic nodules were also present in the testicular parenchyma, suggesting possible associated hematogenous dissemination. The rest of the USG findings in our patient were very typical of chronic granulomatous tuberculous infection.[7–9]

Tuberculous involvement of the epididymides and testes on USG can be of the following types: diffusely enlarged, heterogeneously hypoechoic; diffusely enlarged, homogeneously hypoechoic; nodular enlarged, heterogeneously hypoechoic; or miliary.[7–9] Heterogeneity favors a tuberculous etiology,[8] as in our patient.

Bilateral symmetrical involvement of the epididymides, seen in about 25% of patients,[4] and coarse amorphous calcifications also favor the diagnosis of a chronic granulomatous infection such as tuberculosis. Other findings that were not seen in our patient but can occur in scrotal tuberculosis include thickening of the tunica albuginea or scrotal wall, scrotal abscesses, and cutaneous fistulae.[7] Color Doppler USG shows increased vascularity in the inflamed structures and helps to differentiate infection from infarction. Clinically, it is sometimes difficult to differentiate chronic granulomatous infections or tuberculosis of the epididymides and/or testes from tumor or, rarely, infarction. The ability to differentiate these on USG helps avoid unnecessary epididymo-orchidectomy.
